# Can We Exploit Inflammasomes for Host-Directed Therapy in the Fight against *Mycobacterium tuberculosis* Infection?

**DOI:** 10.3390/ijms25158196

**Published:** 2024-07-27

**Authors:** Lilitha Cebani, Nontobeko E. Mvubu

**Affiliations:** School of Laboratory Medicine and Medical Sciences, College of Health Sciences, University of KwaZulu-Natal, Durban 4000, South Africa; 223145829@stu.ukzn.ac.za

**Keywords:** *M. tuberculosis*, inflammasomes, host response, inflammation, host-directed therapy, gene editing, medicinal plants

## Abstract

Tuberculosis (TB), caused by *Mycobacterium tuberculosis* (*M. tb*), is a major global health issue, with around 10 million new cases annually. Advances in TB immunology have improved our understanding of host signaling pathways, leading to innovative therapeutic strategies. Inflammasomes, protein complexes organized by cytosolic pattern recognition receptors (PRRs), play a crucial role in the immune response to *M. tb* by activating caspase 1, which matures proinflammatory cytokines IL1β and IL18. While inflammation is necessary to fight infection, excessive or dysregulated inflammation can cause tissue damage, highlighting the need for precise inflammasome regulation. Drug-resistant TB strains have spurred research into adjunctive host-directed therapies (HDTs) that target inflammasome pathways to control inflammation. Canonical and non-canonical inflammasome pathways can trigger excessive inflammation, leading to immune system exhaustion and *M. tb* spread. Novel HDT interventions can leverage precision medicine by tailoring treatments to individual inflammasome responses. Studies show that medicinal plant derivatives like silybin, andrographolide, and micheliolide and small molecules such as OLT1177, INF39, CY-09, JJ002, Ac-YVAD-cmk, TAK-242, and MCC950 can modulate inflammasome activation. Molecular tools like gene silencing and knockouts may also be used for severe TB cases. This review explores these strategies as potential adjunctive HDTs in fighting TB.

## 1. Introduction

Tuberculosis (TB), caused by the bacterial pathogen *Mycobacterium tuberculosis* (*M. tb*), continues to stand as one of the most deadly infectious diseases and has accounted for over a billion fatalities in the last two centuries [1]. The global infection rate of *M. tb* is still approximately one in every three people. In the case of newly infected individuals, *M. tb* is successfully eradicated in only 10% of cases, as it has the ability to persist and lie dormant in previous lesions, posing challenges in the effective control of the infection [2].

The interaction between *M. tb* and host receptors triggers the activation of inflammasomes, which are a crucial component of the innate immune system. *M. tb* contains various pathogen-associated molecular patterns (PAMPs) that are recognized by pattern recognition receptors (PRRs) within host cells, such as Toll-like receptors (TLRs), especially TLR2 and TLR4 [3], NOD-like receptors (NLRs), and C-type lectin receptors (CLRs) [3]. Upon recognition of *M. tb* PAMPs, PRRs initiate signaling cascades that activate downstream pathways, including nuclear factor kappa B (NF-κB) and mitogen-activated protein kinases (MAPKs), resulting in the transcriptional upregulation of proinflammatory cytokines, including IL1β and IL18, and the expression of tumor necrosis factor alpha (TNF-α) [4]. Subsequent exposure to *M. tb*-derived components triggers additional signals that activate the inflammasome complex, typically consisting of sensor protein nucleotide-binding leucine-rich repeat receptors (NLRs) or absent in melanoma 2 (AIM2), the adaptor protein ASC (apoptosis-associated speck-like protein containing a CARD), and the effector protein caspase 1. Activation of the inflammasome leads to the cleavage of pro-IL1β and pro-IL18 into their mature forms, which are released from the host cell, promoting inflammation and amplifying the immune response against *M. tb*. This intricate interplay between *M. tb* and host receptors underscores the importance of inflammasomes in host defense against *M. tb* [3].

Canonical inflammasomes, including nucleotide-binding domain-like receptor protein 3 (NLRP3), AIM2, NLR family CARD domain-containing protein 4 (NLRC4) and pyrin, are vital multiprotein complexes in the innate immune response, triggering the maturation and release of proinflammatory cytokines. Conversely, non-canonical inflammasomes activate caspase 11 in mice or caspase 4/5 in humans in response to cytosolic lipopolysaccharide (LPS) from Gram-negative bacteria, leading to gasdermin D (GSDMD) cleavage, pyroptosis, and proinflammatory cytokine release. This pathway provides a rapid response to intracellular bacterial infections and is important for host defense. However, dysregulated activation of non-canonical inflammasomes can contribute to inflammatory diseases [5].

Inflammasomes serve as a platform for the activation of caspase 1, an enzyme that cleaves proinflammatory cytokines, interleukin 1β (IL1β) and IL18, converting them into their active forms during an infection by *M. tb*. These cytokines contribute to an inflammatory response that is critical in the control of *M. tb* during early infection [6]. The dual nature of inflammasome-mediated responses demands careful consideration. While a controlled inflammatory environment is essential for effective host defense during *M. tb* infection, excessive activation can lead to detrimental consequences [7], and hence there is a need for balance in inflammasome modulation to harness its benefits in controlling *M. tb* infection while preventing detrimental inflammatory outcomes. Understanding how *M. tb* manipulates inflammasome activation, and the subsequent impact on the host’s inflammatory response, is important for developing therapeutic interventions [8]. Several studies have exploited different mechanisms of inflammasome activation/suppression during *M. tb* infection, as this can offer novel host-directed perspectives in the control of TB infection [9,10,11]. This review explores these different mechanisms of manipulating canonical and non-canonical inflammasome pathways as novel strategies to fight *M. tb* infections.

## 2. NLRP3 Inflammasome

Infection with *M. tb* is characterized by the presence of granulomatous lung lesions and systemic inflammatory responses. The activation of inflammasomes, crucial multiprotein complexes, plays a critical role in regulating this inflammation, which is essential for both innate and adaptive immunity [6]. The numerous immune cells that contribute to the detection of danger signals, activation of the NLRP3 inflammasome, and subsequent inflammatory responses to combat infection by *M. tb*, tissue damage, and other threats to the host include macrophages, dendritic cells, neutrophils, mast cells, and monocytes. Upon interaction of *M. tb* with these immune cells, an inflammatory response is initiated, which is crucial in shaping the host immune response against the pathogen. Activation of the NLRP3 inflammasome in these cells contributes to the clearance of *M. tb* and the resolution of infection [12]. Collaborating with PRRs, inflammasomes instigate host defense pathways, contributing to the elimination of various viral and bacterial infections, including those induced by *M. tb.* Central to their functionality is the activation of inflammatory caspase 1, a key player in the maturation and release of proinflammatory cytokines, as well as in pyroptosis, which is an inflammatory form of cell death in infected cells that can either be caspase 1- or caspase 11-dependent [7,13].

**Figure 1:**
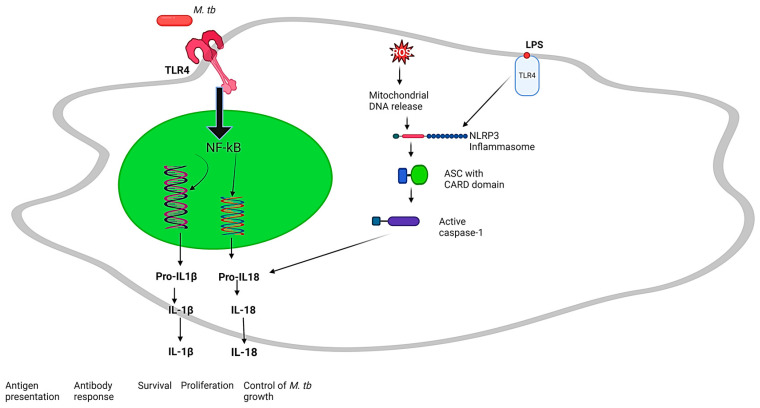
NLRP3 inflammasome activation during infection by *M. tuberculosis*. The NLRP3 inflammasome requires priming before activation, initiated by PAMPs (LPS) binding to host PRRs (TLR4). ASC, the adaptor protein, undergoes ubiquitination and phosphorylation crucial for inflammasome assembly. Upon assembly, the activated inflammasome cleaves pro-caspase 1 into its mature form, which then processes pro-IL1β and pro-IL18 into their active cytokine forms. The interactions between activated NLRP3 inflammasome and ASC via PYD–PYD interactions, and pro-caspase 1 binding to ASC through CARD–CARD interactions has been depicted. Image was created in BioRender.com.

The initial signaling events triggered by PRRs during *M. tb* infection prime the NLRP3 inflammasome. Priming involves the upregulation of NLRP3, pro-IL1β and pro-IL18 expression, and this process sensitizes the inflammasome and prepares it for activation. There are other mechanisms that have been suggested to be involved in the activation of the NLRP3 inflammasome during *M. tb* infection. These mechanisms include the efflux of potassium ions (K+): evidence indicates that potassium efflux precedes the activation of the NLRP3 inflammasome and induces a structural alteration in NLRP3 that promotes its oligomerization [14]. This suggests that targeting K+ efflux pathways could be explored as a therapeutic strategy to either activate or enhance the activation of NLRP3, potentially aiding in the control of intracellular pathogens [14]. Other mechanisms are the disruption of lysosomes and the excess production of reactive oxygen species (ROS), which can be generated by host phagocytes as a defense mechanism against *M. tb* [5]. This can lead to mitochondrial dysfunction and the release of mitochondrial DNA, which has been suggested to be a danger signal that stimulates NLRP3 inflammasome activation. The activation of NLRP3 inflammasome mechanisms during infection by *M. tb* are shown in Figure 1 below. These events can converge to initiate the assembly of the NLRP3 inflammasome complex, which is composed of NLRP3, the adaptor molecule apoptosis-associated speck-like protein containing a CARD domain (ASC), and pro-caspase 1, in order to induce the processing and secretion of IL1β and IL18 cytokines. The recruitment of ASC to NLRP3 facilitates the activation of caspase 1 [5]. Once activated, caspase 1 cleaves the precursor forms of IL1β and IL18 into their active forms, which are then released from the cell (Figure 1).

A study by Rastogi et al. [15] revealed that one of the mechanisms that *M. tb* uses to inhibit the activation of the NLRP3 inflammasome is through its phosphokinase PknF when the *M. tb* serine/threonine PknF inhibits the activity of xanthine oxidase (XO) enzyme, resulting in reduced cytosolic reactive oxygen species (ROS) levels. It also mediates the inhibition of NLRP3 inflammasome activation by preventing potassium (K+) and chloride (Cl-) efflux. The *M. tb* serine/threonine kinase PknF is responsible for the inhibition of NLRP3 inflammasome-dependent IL1β production and pyroptosis [15]. Their study also showed that cells infected with an *M. tb pknF* mutant show increased XO activity compared to those infected with wild-type *M. tb*, leading to higher XO-mediated ROS production.

*M. tb* inhibits NLRP3 inflammasome activation through a mechanism that does not rely on the early secretory antigenic target 6 secretion system-1 (ESX-1), a protein export system that delivers bacterial virulence factors to host cells during infection [15].

## 3. AIM2 Inflammasome

Activation of the AIM2 inflammasome, specifically triggered by *M. tb* infection, is crucial for the host immune response against this pathogen. This inflammasome enhances the adaptive immune response by activating and recruiting antigen-presenting cells, such as dendritic cells, macrophages, and B cells, thereby initiating adaptive immune responses against *M. tb.* AIM2 inflammasome activation during *M. tb* infection initiates the innate immune response, leading to inflammation, elimination of infected cells, and shaping of the adaptive immune response to control and clear *M. tb* infection [7].

**Figure 2:**
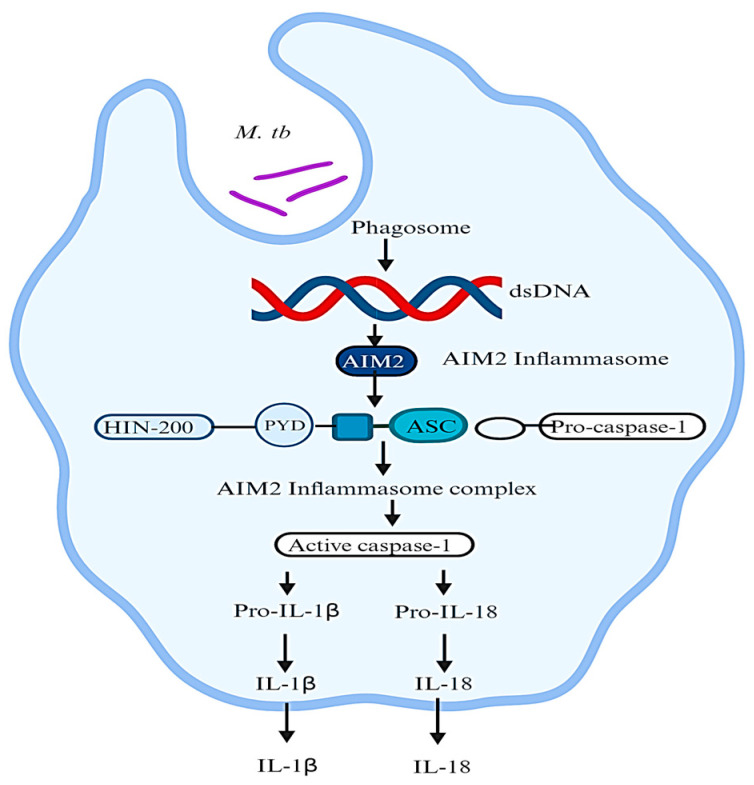
AIM2 inflammasome activation during infection by *M. tuberculosis*. Double-stranded DNA released from bacteria or damaged mitochondria binds to AIM2, inducing assembly of AIM2 inflammasome that consists of AIM2, ASC, and pro-caspase 1. Activated caspase 1 then cleaves pro-interleukin-1β and gasdermins to mediate inflammation and pyroptosis, respectively. Release of bacterial DNA into the cytoplasm relies on bacteriolysis mediated by interferon-inducible GTPases, whose expression is activated by the cGAS–STING axis, also activated by cytosolic double-stranded DNA (dsDNA). Image was created in BioRender.com.

The cytosolic protein AIM2 is equipped with a pyrin domain (PYD) at its N-terminal and an HIN-200 domain at its C-terminal. This unique structure allows AIM2 to act as a sensor for cytosolic DNA and specifically recognize the DNA released by *M. tb* during infection [16]. Recognition of *M. tb* DNA by AIM2 initiates the activation of the AIM2 inflammasome, a multiprotein complex involved in triggering pyroptosis, a type of cell death that promotes inflammation and aids in clearing the infection caused by *M. tb*. AIM2 forms a complex with the adaptor protein ASC (apoptosis-associated speck-like protein containing a CARD) and the effector protein caspase 1 in response to the detection of *M. tb* DNA [17]. This assembly of AIM2, ASC, and caspase 1 leads to the activation of caspase 1 via autoproteolytic cleavage. Activated caspase 1 then initiates the processing and secretion of proinflammatory cytokines IL1β and IL18, which are crucial for mounting an efficient immune response against *M. tb* [16]. Figure 2 depicts the activation of the AIM2 inflammasome during infection by *M. tb.* The release of IL1β and IL18 promotes the recruitment and activation of immune cells, enhancing the clearance of *M. tb* infection. Additionally, these cytokines contribute to the formation of granulomas, which are structures involved in containing the bacterium and preventing its spread [18].

The stimulator of interferon genes (STING) is a DNA sensor located in the endoplasmic reticulum (ER) of immune cells. It plays a crucial role in regulating innate immune responses. STING activation enhances host immunity and is essential for clearing various pathogens, including viruses and bacteria. The cyclic GMP-AMP synthase (cGAS) serves as a DNA recognition receptor that binds to dsDNA from both foreign and self-origin molecules without any sequence specificity [19].

Cytosolic DNA sensor proteins include cGAS and AIM2-like receptor (ALR) inflammasomes, which are AIM2 and IFN-γ-inducible protein 16 (IFI16). Both AIM2 and IFI16 have an HIN200 domain that directly binds to DNA as mentioned above, as well as a pyrin domain.

*M. tb* has evolved mechanisms to inhibit AIM2 inflammasome activation, allowing it to evade immune detection and establish a persistent infection. This AIM2 inflammasome activation inhibition occurs likely by introducing effectors into the host cell cytosol, as this inhibition depends on the ESX-1 secretion system. Mutants of *M. tb* that lack ESX-1 components fail to inhibit AIM2 inflammasome activation, leading to enhanced cytokine production and pyroptosis [20]. Shah et al. [20] also discussed *M. tb* suppressing the secretion of IFN-β in infected cells, which might be a mechanism to inhibit the activation of the AIM2 inflammasome. Thus, the co-secretion of a potential AIM2 inhibitor and IFN-β inhibitor through the ESX-1 secretion system into the host cell cytosol, along with cytosolic *M. tb* DNA, likely plays a crucial role in evasion of AIM2 inflammasome activation by *M. tb*. Its ability to inhibit the AIM2 inflammasome is important for its survival within the host. By inhibiting AIM2 activation, *M. tb* prevents pyroptosis, a type of programmed cell death that restricts bacterial replication and dissemination. Moreover, the suppression of proinflammatory cytokines like IL1β and IL18 enables *M. tb* to evade immune detection and reduces the recruitment of immune cells to the infection site.

Excessive activation of the inflammasomes can cause tissue damage and contribute to chronic inflammatory diseases. Modulating host cell death pathways can be an effective strategy to inhibit inflammasome activation and reduce inflammation during infections. Apoptosis and the recently identified necroptosis represent well-understood forms of programmed cell death, with ongoing research exploring the cross talk between these pathways, particularly mediated by death receptor signaling [21].

Several studies have revealed the importance of early inflammasome activation during *M. tb* infection, as reviewed by [22]. These host inflammatory pathways are involved in the production of essential inflammatory cytokines that can contribute to early immune cell recruitment to the site of infection and bacterial clearance. Inhibition of inflammasome activation is not the only mechanism that can be explored in treating *M. tb* infection: other mechanisms include strategies that encompass conventional antibiotic therapy, immunological approaches, and innovative therapeutic methods. These approaches are designed not only to eliminate the bacteria but also to boost the host’s immune response and control the inflammation-induced damage associated with the infection [23].

## 4. Strategies of Exploiting the Inflammasome in *M. tb* Infection

Approaches to manipulate the inflammasomes during *M. tb* infection encompass a range of strategies directed towards boosting or suppressing its function. Some of the potential strategies include host-directed therapies (HDTs) that can fine-tune the host’s immune response, including the inflammasome, to better control *M. tb* infection while minimizing collateral tissue damage. HDTs seek to regulate the immune system to enhance the effectiveness of macrophage effector mechanisms, minimize inflammation, and mitigate tissue damage resulting from host–pathogen interactions [24]. They may also serve to rebalance the immune response that *M. tb* has so cleverly manipulated. This is an attractive prospect, as it tends not to have a specific target as with antimicrobials, and therefore the risk of developing resistance is minimal.

Their rich bioactive chemical diversity and substantial contribution to drug discovery make medicinal plants a promising avenue for overcoming drug resistance. Additionally, they are acknowledged as valuable reservoirs of highly potent anti-mycobacterial compounds [25]. Some investigations indicate that medicinal plants and their derivatives have the potential to hinder the NLRP3 inflammasome at both transcriptional and post-transcriptional stages in in vitro and in vivo *M. tb* infection models. This suggests that these plants and their bioactive derivatives could be explored as host-directed adjuvants with anti-TB properties, aiming to control the inflammatory response induced by *M. tb* [26].

Several medicinal plants have been studied for their anti-TB effects, some of which exhibit properties that may help minimize inflammation through modulation of the NLRP3 pathway. While specific mechanisms may vary, these plants often contain bioactive compounds with anti-inflammatory properties. These include silymarin [27], aloe emodin [28], silibinin (SB) [27], also called silybin, a natural polyphenolic flavonoid from milk thistle (*Silybum marianum*) that has been utilized as an anti-inflammatory herb and hepatoprotective agent. This was observed when SB was administered to differentiated THP-1 cells for two hours and reduced caspase 1 autocleavage induced by the activation of the NLRP3 inflammasome using LPS and ATP, as reviewed by Peng et al. [29]. SB demonstrated protective effects against LPS-induced acute lung injury in mice by inhibiting the NLRP3 inflammasome and NF-κB signaling, despite not affecting the protein levels of NLRP3, ASC, and caspase 1. Artemisinin, derived from *Artemisia annua*, exhibits anti-TB activity and synergizes with conventional TB drugs, potentially aiding in combating drug-resistant TB strains. Traditional uses of *Artemisia* species, including *A. annua* and *A. afra*, for treating fever and cough align with their therapeutic exploration against pathogens like mycobacteria. *A. annua*, particularly known for producing artemisinin, demonstrates anti-tubercular properties, with both artemisinin and its derivative artesunate showing efficacy against *M. tb* in vitro and in rat-infected models [30].

Utilizing medicinal plants to modulate immune system components represents a potential and promising approach in managing *M. tb* infections. This strategy avoids direct targeting of the bacillus, mitigating the pathogen’s inclination to develop drug resistance and encounter selective pressure [26]. Consequently, medicinal plants offer an innovative perspective in host-directed intervention for controlling immunopathology within the alveoli. This intervention involves inhibiting the inflammasome pathways, which play a pivotal role in the production of IL1β and IL18 cytokines during infections by *M. tb.*

Using medicinal plants to harness the inflammasome in *M. tb* infection provides numerous benefits. These include deriving bioactive compounds naturally, lowering the likelihood of drug resistance enhancing accessibility and affordability, ensuring safety, aligning with cultural practices, offering potential as adjunct therapies, and promoting sustainability [31]. Despite the beneficial properties of medicinal plants and their bioactive derivatives of host inflammasome pathways, many authors argue about the safety aspect of these molecules, since they have the potential of worsening the individual’s health if not extensively tested and approved by regulatory committees [32,33].

Current clinical treatments for NLRP3-related diseases primarily involve medications targeting IL1β, such as anakinra, canakinumab, and rilonacept. However, there are concerns regarding their potential to increase infection risks. Consequently, inhibitors directly targeting the NLRP3 inflammasome may offer greater efficacy in managing NLRP3-driven diseases like TB. Several small-molecule inhibitors, including OLT1177, INF39, CY-09, JJ002, and Ac-YVAD-cmk, show promise in this regard [34]. TAK-242, or resatorvid, is one of the small-molecule inhibitors that selectively targets TLR4 signaling. By binding to the intracellular domain of TLR4, it inhibits its interaction with adaptor proteins like MyD88 and TRIF, crucial for downstream signaling cascades. This action effectively suppresses the production of proinflammatory cytokines such as TNF-α, IL1β, and IL6, leading to reduced inflammation. TAK-242 has shown therapeutic potential in various inflammatory conditions, including sepsis, rheumatoid arthritis, and inflammatory bowel disease. Notably, it can modulate excessive inflammation without inducing immunosuppression, making it an attractive candidate for treating inflammatory disorders [35]. Another inhibitor is BAY 11-7082, which acts by inhibiting the activation of NF-κB, a transcription factor involved in regulating the expression of many proinflammatory genes. Specifically, BAY 11-7082 blocks the phosphorylation and subsequent degradation of inhibitor of kappa B (IκB), a protein that normally sequesters NF-κB in the cytoplasm. By preventing IκB degradation, BAY 11-7082 prevents NF-κB from translocating to the nucleus and activating the transcription of proinflammatory genes. As a result, BAY 11-7082 effectively suppresses the production of various proinflammatory cytokines and mediators, including TNF-α, IL1β, and iNOS [36].

**Figure 3:**
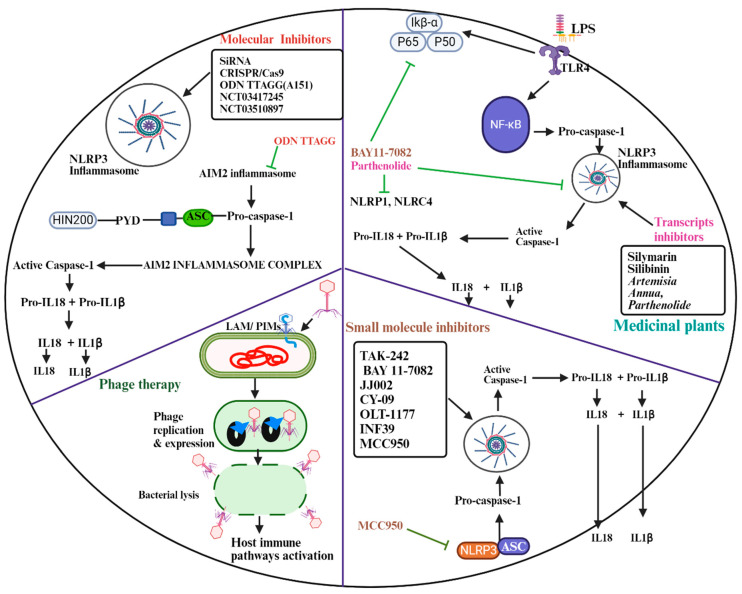
Small molecules, medicinal plants and/or bioactive derivatives, molecular tools and phage therapy are a few strategies for manipulating inflammasomes during infection by *M. tb*. The inhibitors in Figure 3 (not limited to) block inflammasome-induced responses during infection and are specific to inflammasome pathways targeted. Image was created in BioRender.com.

Several small molecules have shown promise in exploiting inflammasome responses during *M. tb*. These compounds target specific components of the inflammasome pathway, offering potential therapeutic avenues. MCC950 is another example of a specific NLRP3 inhibitor that effectively inhibits NLRP3 by blocking the ATPase activity of NLRP3, thus impeding inflammasome assembly and subsequent IL1β production (Figure 3) [37].

The utilization of molecular tools such as gene-editing technologies, gene-silencing techniques, natural compounds, and peptide inhibitors offers a sophisticated and targeted approach to manipulate inflammasome responses during *M. tb* infections. Gene-silencing techniques, particularly RNA interference (RNAi), such as small interfering RNA (siRNA), can be effectively exploited as molecular tools for HDT against inflammasomes during infection by *M. tb.* SiRNA can be designed to target specific components of the inflammasome pathway, such as NLRP3 or ASC, which are crucial for inflammasome assembly and activation. By silencing the expression of these inflammasome components, siRNA can prevent the formation of functional inflammasomes, thereby attenuating the production of proinflammatory cytokines in response to infection infections [38], which remains to be validated in *M. tb* infection. Researchers have engineered various molecular tools, including genome-editing nucleases such as CRISPR/Cas9, transposons, episomes, and siRNA/shRNA. These tools are introduced into the host’s cells in the form of DNA, RNA, or protein utilizing physical or biochemical techniques [39]. The latest siRNA to receive approval is Amvuttra (vutrisiran). Other siRNA products are currently undergoing testing, including clinical trials of fitusiran (NCT03417245) in phase III, which has shown the potential clinical application of siRNAs. Teprasiran (phase III—NCT03510897) and cosdosiran (phase II/III—NCT02341560) are two other siRNAs being explored for HDT through gene silencing [39]. Teprasiran targets p53-mediated cell death, which is also favored by the NLRP3 inflammasome [40], and therefore this molecule holds great potential in exploration for TB-related lung tissue damage. Lastly, the current siRNA tools that are approved and those undergoing clinical trials have great potential for being explored for host inflammasome genes during *M. tb* infection and thus remain to be explored for this notorious pathogen.

Gene-editing technologies enable the manipulation of genes, facilitating the insertion of permanent and precise DNA modifications into the genome. There are four primary types of gene-editing nucleases employed in research: meganucleases (MNs), zinc finger nucleases (ZFNs), transcription activator-like effector nucleases (TALENs), and CRISPR/Cas9 [41]. While MNs, ZFNs, and TALENs rely on protein–DNA interactions to target specific sites, CRISPR/Cas9 systems utilize RNA–DNA interactions to guide them to the target site. CRISPR/Cas9 nucleases offer several advantages over other gene-editing nucleases, notably the simplicity of guide RNA (gRNA) design. CRISPR/Cas9 technology has the potential to manipulate inflammasomes in *M. tb* infection by targeting specific genes involved in the inflammasome pathway. Researchers design gRNAs complementary to sequences within genes encoding components of the inflammasome complex, such as NLRP3 or AIM2, and deliver them along with the Cas9 enzyme into host cells infected with *M. tb* [39]. Upon delivery into these cells, the CRISPR/Cas9 system induces double strand breaks at the target sites, resulting in gene disruption or knockout. This disruption hinders the assembly or activation of the inflammasome complex, thereby altering the host immune response to *M. tb* infection.

Another CRISPR/Cas system based on Cas13a, an RNA-guided RNA-targeting endonuclease, holds great promise as a molecular diagnostic platform. The discovery of this CRISPR/Cas system has generated a wave of development of innovative diagnostics that take advantage of both the sensitivity of PCR amplification and the specificity of the CRISPR system [42]. CRISPR-*M. tb* test has demonstrated superior diagnostic performance compared to traditional methods like Xpert and culture [43]. This superiority not only stems from its heightened sensitivity but also from its requirement for less sample input and shorter turnaround time. However, it is worth noting that this method may not be able to detect certain strains of *M. tb* lacking IS6110 genomic segments. In settings where resources are limited, an alternative test utilizing PCR-CRISPR/Cas13a might prove more practical, especially for diagnosing pediatric TB and tuberculous meningitis patients, despite the slightly longer turnaround time associated with DNA-targeting CRISPR-Cas systems [42].

Genome-editing nucleases are utilized to disable specific target genes like IL13Ralpha2, PD-1, CISH, TRAC, and B2M. CRISPR/Cas9 technology in particular has gained prominence in clinical trials, with a focus on solid tumors and hematological malignancies [39]. In solid tumors, CRISPR/Cas9-mediated knockouts of PDC1 and TRAC have been investigated (NCT02793856, NCT03081715, NCT03044743, NCT03545815, NCT03747965) to alleviate exhaustion in CAR-T cells. For hematological malignancies, genes such as TRAC, B2M, CD7, CD28, CD19, CD20, and CD22 have been targeted using CRISPR/Cas9 (NCT03190278, NCT03166878, NCT03398967, NCT03690011), depending on the specific characteristics of the cancer. However, a significant drawback of these gene-editing nucleases is their potential for off-target effects, which can result in mutagenesis and translocations [39], and these should be tested for their potential knockout of the inflammasome-related genes. Combining these molecular tools with a thorough understanding of inflammasome biology holds great potential for the development of innovative therapeutic strategies that can modulate host responses and enhance immune-mediated control of *M. tb* infections (Figure 3).

Among the array of emerging antibacterial strategies, phage therapy also emerges as a promising and precise solution. Mycobacteriophages, which are viruses targeting bacteria like those within the *Mycobacterium tuberculosis* complex (MTBC), hold significant potential as antimicrobial agents [44]. Both phages and protein-derived phages show promise in combating bacterial infections. Combinations of phages in cocktails can enhance the spectrum of bacteria that they can lyse. Chegini et al. [45] have demonstrated the effectiveness of combining antibiotics with phages to combat bacterial infections. However, there are notable limitations and concerns associated with phage therapy, including the human immune response to phages, the potential transfer of antibiotic resistance genes, the emergence of resistance to phages, and safety considerations.

One potential approach is to engineer mycobacteriophages to deliver genetic cargo that can interfere with inflammasome activation or function within infected host cells. This may entail delivering nucleic acids containing inhibitors targeting specific inflammasome components or regulators, thereby attenuating exaggerated inflammation and enhancing the host’s defense mechanisms against *M. tb* [46]. Mycobacteriophages can also be engineered to specifically attack *M. tb* residing within host cells, resulting in bacterial cell lysis and the release of antigens capable of eliciting immune responses. Such a mechanism could potentially activate host immune pathways, including the inflammasome, to enhance the clearance of *M. tb*-infected cells and limit bacterial dissemination (Figure 3) [47]. Significant challenges remain in the development and application of mycobacteriophage-based therapies. Although phage–antibiotic combination presents a promising avenue for treating bacterial infections, it comes with its own set of challenges and limitations. One significant issue is the potential for interactions between antibiotics and phages. Antibiotics might impede phage replication, diminishing the effectiveness of phage therapy. Conversely, phages may also interfere with antibiotic activity. Thus, careful selection of antibiotics and phages, along with optimizing treatment timing and dosage, is essential to maximize phage–antibiotic combination efficacy [44]. There is concern regarding the development of resistance to both antibiotics and phages. While phage–antibiotic therapy can help mitigate the emergence of phage-resistant bacteria, antibiotic usage may elevate the risk of antibiotic resistance. Hence, ongoing monitoring for resistance and the development of strategies to mitigate this risk are imperative [44,48].

The discovery of molecular tools has paved the way for a versatile approach to treating various diseases. Despite the considerable potential of these tools for modulating inflammasome responses during *M. tb* infection, several challenges hinder their clinical implementation, and it is essential to consider the importance of specificity and safety to mitigate off-target effects and toxicity. Additionally, the high costs associated with these tools’ development and production pose a barrier to their accessibility, particularly in resource-limited regions where TB is most prevalent. Encouraging outcomes have been observed in numerous preclinical investigations and clinical trials, particularly for CRISPR/Cas9. Nevertheless, the safe and efficient delivery of genome-editing components remains a crucial hurdle for in vivo genome-editing therapy. Adeno-associated virus (AAV) is one of the most commonly used vector systems to date, yet challenges persist regarding its immunogenicity towards the capsid, liver toxicity at elevated doses [38], and the potential genotoxicity stemming from off-target mutagenesis and genomic integration. Recently introduced transient delivery platforms, such as virus-like particles and lipid nanoparticles, hold promise in addressing certain drawbacks associated with AAV.

## 5. Conclusions and Future Perspectives

The exploration of inflammasomes as a potential HDT for combating *M. tb* infection presents a promising avenue in the quest for more effective TB treatment. The intricate interplay between *M. tb* and the host immune response underscores the pivotal role of inflammasomes in shaping the inflammatory background during infection. As mentioned above, targeting inflammasomes offers a tantalizing prospect of fine-tuning the host immune response, enhancing pathogen clearance while minimizing immunopathological consequences. By modulating inflammasome activity, researchers aim to harness the innate immune system’s ability to fight *M. tb* without inducing excessive inflammation or tissue damage, which is usually detrimental to the host.

The potential use of medicinal plants, molecular tools, and small molecules in modulating inflammasome activity highlights the growing interest and investment in this area of research. However, translating these promising findings into clinically effective therapies poses significant challenges. Despite these challenges, the exploration of host signaling pathways such as inflammasomes as an HDT holds considerable promise for revolutionizing TB treatment. With continued research and innovation, inflammasome-targeted therapies will offer new avenues for fighting *M. tb* infection, ultimately contributing to the global effort to control and eradicate TB.
